# Engineering of PVA/PVP Hydrogels for Agricultural Applications

**DOI:** 10.3390/gels9110895

**Published:** 2023-11-12

**Authors:** Eyal Malka, Shlomo Margel

**Affiliations:** Bar-Ilan Institute of Nanotechnology and Advanced Materials (BINA), Department of Chemistry, Bar-Ilan University, Ramat-Gan 5290002, Israel

**Keywords:** PVA/PVP, agricultural applications, PVA/PVP-based hydrogels, controlled release, sustainability, biodegradability

## Abstract

Hydrogels have gained significant popularity in agricultural applications in terms of minimizing waste and mitigating the negative environmental impact of agrochemicals. This review specifically examines the utilization of environmentally friendly, shapable hydrogels composed of polyvinyl alcohol (PVA) and polyvinylpyrrolidone (PVP) in various casings for crop protection against different pests, fertilizing, and watering. To activate their effectiveness, PVA/PVP hydrogels were loaded with both hydrophilic and hydrophobic environmentally friendly pesticides, namely hydrogen peroxide (HP), the essential oil thymol, and urea as a fertilizer, either separately or in combination. This review covers various physical and chemical approaches used for loading, shaping, and controlling the release profiles of pesticides and fertilizers. Additionally, it explores the evaluation of the chemical composition, structure, classification, rheology, and morphology of the hydrogels as well as their impact on the thermal stability of the encapsulated pesticides and fertilizer, followed by biological tests. These hydrogels significantly contribute to the stabilization and controlled release of essential nutrients and biocides for plants, while maintaining excellent biocidal and fertilizing properties as well as sustainability characteristics. By shedding light on the latest insights into the concepts, applications, and results of these hydrogels, this review demonstrates their immense potential for widespread future use in agriculture.

## 1. Introduction

A hydrogel is characterized by a porous three-dimensional (3D) framework composed of hydrophilic polymers that can undergo swelling, effectively accommodating a large water content. Swollen hydrogels are sustained by chemical or physical cross-linking among constituent polymer chains, ensuring structural integrity. Qualification as a hydrogel requires that water constitutes at least 10% of the total weight (or volume). Hydrogels are systematically categorized into distinct branches. PVA/PVP hydrogels are constructed from two synthetic, nonionic, homopolymer chains that undergo physical or chemical cross-linking [1,2,3]. An extensive investigation was conducted on a physically cross-linking (via hydrogen bonds) PVA/PVP hydrogel. Gelation, for example, is initiated by freezing followed by thawing [4,5,6]. During this process, a phase separation occurs; the polymeric chains get closer to each other, forming denser polymer regions connected by entangled hydrogen bond interactions, resulting in a strengthened structure. Meanwhile, water migrates from different regions, forming separate water phases. Repeated freeze–thaw cycles enhance a hydrogel’s mechanical properties [7,8,9]. Strengthened hydrogels restrict the release of entrapped active molecules, significantly influencing release behavior [10,11]. The PVA/PVP ratio directly impacts various parameters, such as water content, absorption capacity, transmittance, and tensile strength [4]. Viscosity decreases with temperature, eventually leading to melting; therefore, it is reversible [12,13,14]. Hydrogen bonds are susceptible to heat. Upon reaching the boiling point of water, they dissolve back into their initial precursor solutions [15,16,17,18]. From a sustainability perspective, this holds significant implications, as the polymers can be reused, resulting in reduced waste that would otherwise accumulate in the environment. Yet, physical cross-linking alone does not always confer the requisite strength.

An alternative method for fabricating PVA/PVP hydrogels involves cross-linking by irradiation. Many studies effectively showcase the utilization of gamma and ultraviolet irradiation, as well as high-voltage electron beams, as viable means for cross-linking PVA [19,20] and PVP [21,22]. Some instances were documented where these methods were applied to create cross-linked PVA/PVP hydrogels [23,24,25,26]. The mechanism underpinning these approaches is rooted in radical formation through homolytic cleavage, leading to the creation of reactive sites. These radical sites propagate with other polymer chains, giving rise to the formation of robust covalent cross-linking that contributes to enhanced strength [27,28,29]. The degree of cross-linking is contingent upon factors such as the radiation source, its intensity, and exposure duration. Hydrogels subjected to irradiation exhibit distinctive features, including reduced pore size, heightened mechanical strength, diminished swelling capacity, and an extended release profile for encapsulated active molecules [23]. Nonetheless, it is important to note that irradiation can potentially result in degradation or adverse environmental effects on substances entrapped within hydrogels. For instance, the pesticide thymol and hydrogen peroxide (HP) were shown to be susceptible to the effects of irradiation [30,31]. 

Another avenue for covalent cross-linking involves a chemical approach, wherein a cross-linker covalently connects polymeric chains. Diverse types of linkers are utilized for PVA/PVP hydrogels. Prominent examples include formaldehyde, glutaraldehyde (GA), and derivatives, which have been extensively studied and their utility has been showcased in various domains, such as biomedical devices, biological applications, and industrial settings [32,33,34,35]. The cross-linking mechanism relies on the hydroxyl groups of PVA initiating nucleophilic attacks on the electrophilic aldehyde or derivative groups of GA. This process results in the formation of acetals, contributing to the establishment of covalent cross-linking [36,37]. The incorporation of these new acetal groups, derived from the cross-linker, significantly enhances strength and stability. The obtained hydrogel is inert, particularly in neutral conditions, unaffected by acidic environments [38,39]. The chemical cross-linking process can potentially introduce certain undesired side effects. The cross-linker might react with the encapsulated substances, forming byproducts [40,41], or unreacted residues of the cross-linker can persist, posing a toxic risk [42]. Furthermore, the outcome of this process is covalent bonds, which are inherently nearly irreversible [43,44].

Owing to their distinctive characteristics, hydrogels were identified as suitable for a wide range of applications. These include drug delivery, the creation of contact lenses, development of scaffolds for tissue engineering, wound healing, functional tissue generation, capturing dyes and heavy metals, pH and biosensors, supercapacitors, and spinal cord regeneration by injection [18]. Simultaneously, hydrogels gained recognition in agricultural contexts, primarily due to their capacity to serve as carriers for pesticides, fertilizers, and water. Their ability to retain these substances and facilitate extended, controlled release within the plant environment contributed to their increasing utilization in agriculture [45,46,47,48,49,50]. The outcome is a surplus availability of nutrients and effective irrigation while concurrently minimizing pest-related challenges. Irrigation stands as an imperative prerequisite for ensuring the viability of plants. Nevertheless, conventional direct watering can lead to substantial water loss owing to evaporation and rapid infiltration, pronounced particularly in desert climates and areas with low soil density, respectively. Hydrogels exhibit exceptional water retention capabilities due to their hydrophilic nature and expansive porous structure. This unique composition allows them to accumulate significant water volumes, with the capacity for prolonged retention and gradual controlled release. As a result, incorporating water-laden hydrogels into plant soil facilitates efficient management of soil moisture.

Extensive research has been conducted on PVA and PVP hydrogels utilized individually as additives in soil conditioners [51,52,53]. However, there is a scarcity of relevant works in the literature regarding utilization of a combination of PVA and PVP in hydrogel-based soil conditioners. Despite their potential, these polymers still encounter several challenges. From a cost–benefit perspective, the established polyolefin plastic industry remains dominant due to its cost-effectiveness and product durability [54,55,56]. Owing to the high water uptake and release characteristics of PVA/PVP, these materials are susceptible to swelling and shrinking, leading to dimensional changes [57,58,59]. Protection against pests is an additional critical factor, as plants and crops are susceptible to pest attacks and damage. Diverse pesticides were formulated into these hydrogels to mitigate such challenges [60,61,62]. However, many pesticides are regarded as toxic and exhibit gradual degradation over time [63,64]. Consequently, the residues they leave behind have the potential to inflict harm upon both consumers and the environment. As a result, significant endeavors are undertaken to explore alternative eco-friendly solutions. As an example, consider HP, recognized for its hydrophilic nature and strong oxidizing properties, which impart well-established virucidal and fungicidal activities. Importantly, it also mitigates toxic effects on crops. In addition, as HP naturally breaks down into water and oxygen over time, there is no concern about toxic residues impacting the environment [10,65,66].

Another example is thymol, a natural essential oil terpenoid with hydrophobic properties extracted from *Thymus vulgaris*. Thymol was proven effective against a wide range of pests, including microorganisms and insects. Importantly, it is not classified as an environmental toxin and does not pose a risk to human health [67,68,69]. Furthermore, it undergoes decomposition over time through hydrolysis and aqueous photolysis in normal processes [70]. Regarding fertilizers encapsulated within hydrogel carriers, it is important to note that N, P, and K constitute the three pivotal nutrients that are essential for plant growth [71,72,73]. These nutrients are accessible in the form of synthetic or natural compounds, with the former offering greater efficiency and the latter providing environmentally friendly advantages [74,75,76,77]. However, integrating natural fertilizers into a hydrogel casing establishes a barrier that retains and gradually releases them into the environment. The outcome is an extended release that facilitates an efficient and controlled supply of natural nutrients to plants [78,79].

Several studies showcase the utilization of PVA and PVP combinations in coatings and membranes designed for controlled release of fertilizers [80,81]. Nonetheless, the task of developing environmentally sustainable hydrogels involves engineering adaptable physical and/or chemical cross-linking properties, which must be achieved while ensuring that the hydrogel retains its mechanical integrity to maintain consistent shape and compatibility with both hydrophobic and hydrophilic active compounds. A hydrogel’s composition should consist of components that are officially approved for agricultural and post-harvest applications. The amalgamation of these stipulations presents a multifaceted challenge. For this purpose, several promising materials were selected. PVA holds promise as a constituent for several reasons. It is a water-soluble hydrophilic polymer possessing favorable attributes including being non-toxic, non-corrosive, biocompatible, easily processable, and well regarded in pharmaceutical applications. These applications encompass a range of uses, from cartilage replacements and contact lenses to wound healing and eye drops. It also finds extensive application in the cosmetic industry, including lotions, creams, makeup, hair treatments, and styling aids.

Similarly, PVA plays an important role in the food industry as thickener and moisture barrier. Notably, it is recognized and approved by the FDA as food additive E1203 [82,83,84,85,86,87]. Furthermore, it is considered environmentally friendly due to its biodegradable nature [88,89,90]. Many studies were conducted on PVA in hydrogel form, all of which strongly substantiated its suitability for applications in agriculture and the food industry [91,92,93]. Another promising component, PVP, serves as a highly compatible polymer alongside PVA, owing to its non-toxic nature and FDA approval for food applications. It is recognized as food additive E1201 and employed as an emulsifying, stabilizing, and thickening agent [94,95,96]. PVP also plays a significant role in pharmaceutical applications, serving as a plasma volume expander, a binder in pharmaceutical tablets, and a wetting agent, and in dental care products, particularly in tooth-whitening gels. However, PVP distinguishes itself by its distinct chemical properties, enabling effective interactions and carrying both hydrophilic and lipophilic substances. Its advantageous capacity to adsorb substantial quantities of water [97,98] allows PVP to function as a versatile carrier for hydrophilic and hydrophobic fertilizers, pesticides, and water. Many studies present solutions for protection against various pests. Some cases include hydrogels encapsulated with hazardous pesticides [30,31], while others demonstrate more friendly encapsulated substances [47,57]. However, none of the above approaches can be described as constituting a versatile carrier of hydrophobic and hydrophilic substances with the capability to design the bulk material into versatile shapes as capsules, seedling plates, coatings, and beads. Moreover, none of the approaches are officially approved materials for food use. Our review focuses on PVA/PVP-based hydrogels with the capability to achieve optimal strength, water content, and absorption while minimizing transmissivity. These hydrogels are also designed to be shapeable [4] and are adapted for potential agricultural applications. They are loaded with HP and/or thymol and maintain neutral physiological pH conditions, showcasing a wide range of pesticides, and can be shaped into conceptual forms including coatings, seedling plates, capsules, and encapsulated granules or diluted to become sprayable liquids. Hydrogel shapes are reinforced through physical or chemical cross-linking. The emphasis is placed on the physical and chemical interactions within the hydrogel structures and their impact on morphology, the stability of encapsulated substances, and subsequent release behavior. In this review, patterns of pesticides and fertilizers released from PVA/PVP hydrogels and derivatives were examined, considering those that are physically cross-linked, and cases with chemical cross-linking were explored. This evaluation encompasses the study of exchange effects and involves a direct comparison between these two methodologies. Our investigation culminates with contemporary practical implementations of hydrogels in safeguarding crops and plants as well as fostering their growth through protective and fertilization functions.

## 2. Structural Chemistry and Classification of Agricultural PVA/PVP-Based Hydrogels

The fundamental structure of PVA/PVP hydrogels can be achieved through the establishment of physical hydrogen bonds between distinct homopolymer chains [99,100,101]. PVP acts as a highly effective proton acceptor [97,102,103], while PVA serves as a proton donor [104]. The more complex structure involves chemical cross-linking, e.g., via glutaraldehyde, leading to the development of copolymer chains along the backbone or the formation of acetals through interactions involving side chains, introducing possibly more rigid structures [105,106,107]. However, both structures serve as excellent carriers and retainers of water for plants, owing to their hydrophilic side groups. These structures can be loaded with hydrophilic fertilizer and pesticide compounds, such as urea and HP, respectively, as well as hydrophobic ones, like thymol.

**Figure 1:**
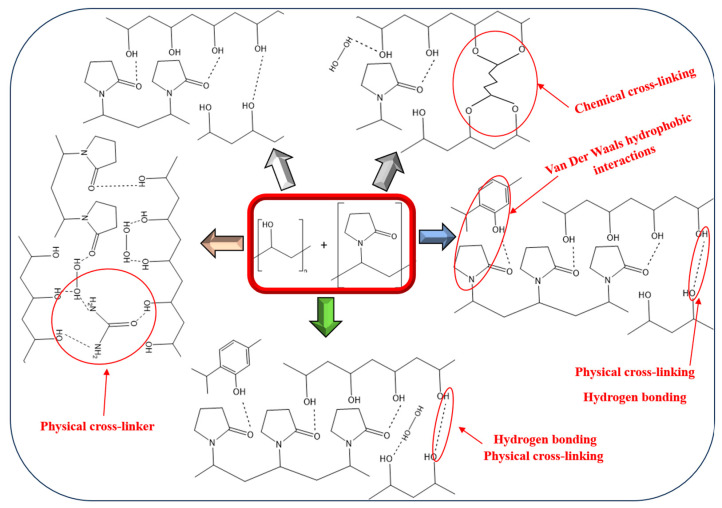
Representative agricultural PVA/PVP hydrogel structures: PVA and PVP precursors (in the red frame), physically cross-linked unloaded PVA/PVP (white arrow) and entrapped with HP and urea (peach arrow), PVA/PVP/thymol (blue arrow), PVA/PVP/HP/thymol (green arrow), chemically cross-linked PVA/PVP/HP (grey arrow) hydrogels.

In the scenario of hydrophilic loaded substances, their retained and slow-release forms are primarily established through branched hydrogen bond interactions between the polymeric caging structure of the hydrogels and these solutes. For instance, urea fertilizer solute comprises amide groups, functioning as a physical cross-linker, while its carbonyl head contributes to additional physical interactions regarding HP pesticide solute, which has the capacity to engage in hydrogen bonding with PVA and form complexes with PVP [108,109]. In the context of thymol, Van der Waals hydrophobic interactions are feasible mainly with PVP due to thymol’s hydrophobic terpene side. Additionally, its hydroxyl group stemming from the phenolic hydrophilic side can participate in hydrogen bonding interactions (Figure 1 blue arrow). In scenarios involving the loading of multiple substances, the possibility of additional interactions between them arises. For instance, the coexistence of urea with HP can result in the formation of a complex [110]. Additional potential hydrogen bond interactions can take place between thymol and urea [111]. However, given that HP is recognized as a distinct oxidizer, the presence of thymol could potentially lead to certain oxidative side reactions [112].

## 3. Hydrogels as Carriers and Controlled Release of Pesticides, Fertilizers, and Water

### 3.1. PVA/PVP Hydrogel Carriers

**Figure 2:**
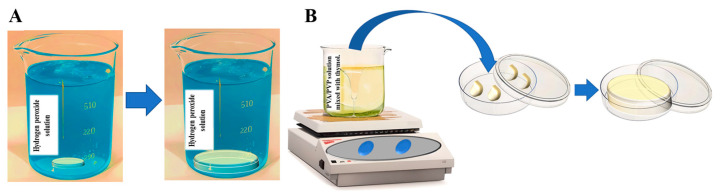
Loading of active substances can occur either through (**A**) the swelling of hydrophilic and heat-sensitive materials or (**B**) the in situ entrapment of hydrophobic and more thermally stable substances.

Hydrogels were modified to transport both water-loving) hydrophilic (and water-repelling (hydrophobic) pesticides and fertilizers [10,65]. This versatility is primarily attributed to the binding capabilities of PVP with a wide range of substances [97]. Loading of active materials can occur by two pathways. One involves a heated hydrogel precursor solution, wherein the active ingredients are mixed, and the mixture is cured into its ultimate form. This method is referred to as the in situ method. The second pathway involves creating a pure hydrogel, which is then dried to remove water. The dried product is immersed in an aqueous solution containing the active ingredient at room temperature. Over time, the hydrogel absorbs the solution and swells until equilibrium is reached. This is referred to as the swelling method (Figure 2A).

The suitability of the loading method depends on the specific properties of the intended substance. The swelling method is particularly suitable for loading hydrophilic and less thermally stable ingredients, such as HP, which is miscible in aqueous solutions and undergoes decomposition when exposed to heat [113]. Consequently, during swelling, HP can readily permeate the hydrogel. Given that this process takes place at room temperature, the pesticide maintains its stability. The active substance’s swelling rate within the hydrogels is a parameter that must be considered. Conversely, hydrophobic and more heat-resistant pesticides such as thymol are better suited for the in situ method. This is due to thymol’s ability to create a uniform and stable mixture, resembling a lotion when combined at moderate temperatures. Nonetheless, when thymol is exposed solely to water at room temperature, it forms a separate oil phase and does not undergo swelling within the hydrogel [114,115]. Recent studies effectively demonstrate the capacity of these hydrogels to carry significant amounts of pesticides and fertilizers, while maintaining their ability to absorb water [10,81,116,117,118].

### 3.2. Controlled Release by PVA/PVP Hydrogel

Upon saturating a hydrogel with water, pesticide, or fertilizer, cargo is released by various pathways when the target comes into direct physical contact with the surface of the hydrogel. Subsequently, the active solutes or colloids undergo diffusion and directly adhere to the roots, foliage, crop shells, or pests of plants. Release is facilitated by a method involving direct contact. Another approach involves situating the hydrogel near its target with a controlled air gap. Over time, this arrangement enables gradual release of pesticides or moisture vapor, establishing a localized environment around the plant. This indirect method enhances plants’ survivability and, correspondingly, their prosperity. Each approach encompasses advantages and disadvantages. On the one hand, direct contact efficiently conveys the released substances to their intended destination, resulting in relatively minimal loss during release. On the other hand, subjecting plants or crops to direct, unbuffered doses of water, nutrients, or pesticides can lead to phytotoxic effects and potential harm, including destruction. The indirect approach mitigates these severe side effects. Nonetheless, while this refined method helps alleviate issues, it may not always yield perfect results. Hence, the choice of the optimal method relies on the inherent resilience of the plant or crop during its exposure as well as the effectiveness of the released substances, all within the context of the specific operational conditions.

**Figure 3:**
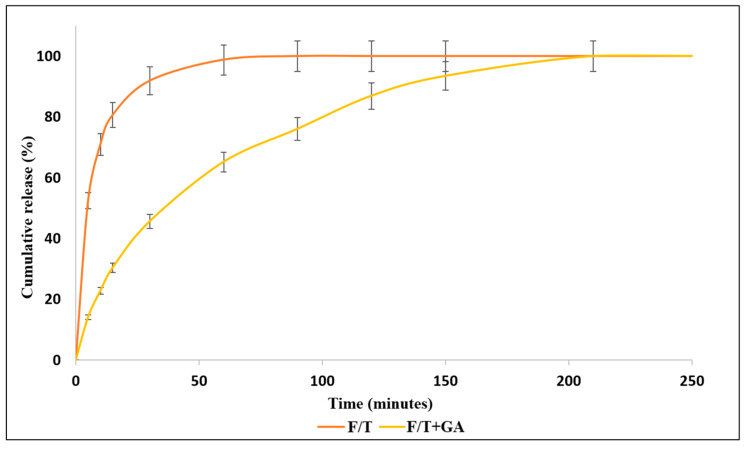
Release rate of HP from PVA/PVP/HP hydrogels to water. Prepared by 3 freeze–thaw cycles (orange) or followed with surface chemical cross-linking by GA (yellow).

Our recent investigations encompass the release of HP pesticide through both direct and indirect methods. The HP release profile exhibited a dependency on the quantity of freezing–thawing cycles undergone by the physically cross-linked PVA/PVP hydrogels. The augmentation in cycles led to extension and modulation of the release [10]. Hydrogels containing a blend of HP and thymol enabled a swifter release of HP. This could be attributed to the repulsive interactions occurring between these distinct types of molecules [65]. A novel aspect of our ongoing research reveals the reinforcement of a HP-entrapped hydrogel through chemical cross-linking utilizing glutaraldehyde under mild acidic conditions. This cross-linking process led to a significantly prolonged release duration in comparison to a previous physical technique (Figure 3). This development holds significant potential for providing plants and crops with sustained, long-term essential supplies. Nonetheless, the presence of unreacted glutaraldehyde residues poses potential risks to both human health and the environment. Consequently, further research is imperative in addressing the issue of residual glutaraldehyde (GA) within loaded hydrogels, all while ensuring the retention of loaded active substances.

**Figure 4:**
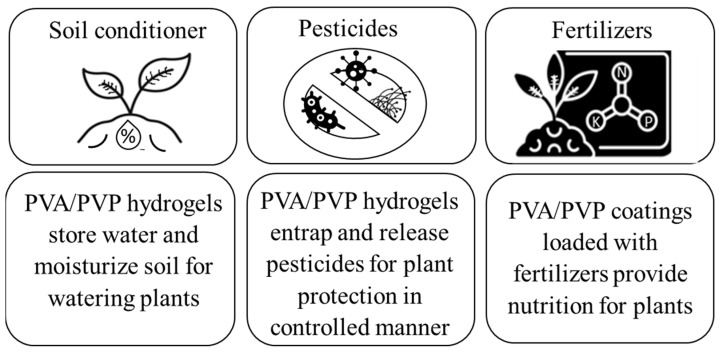
Graphical illustration of the main current agricultural uses of PVA/PVP hydrogels, including coatings, and their future potential as soil conditioner additives.

In the domain of fertilizer release and water retention, an investigation centered on the encapsulation of urea granules [119] using PVA/PVP coatings unveiled significant insights. The encapsulated urea granules displayed a controlled and extended release of urea relative to the fast loss exhibited by uncoated counterparts [117]. These findings are consistent with our investigations pertaining to the controlled release of pesticides [10,65]. Furthermore, the amalgamation of Superabsorbent Polymers (SAP) with either PVA or PVP, when integrated with soil and subsequently irrigated with a precisely measured water volume, resulted in a substantial improvement in water retention. This improvement was notably superior to the water retention observed in untreated control soil conditions [119,120,121,122,123,124,125,126]. This suggests that in prospective advancements of soil conditioners, the incorporation of PVA/PVP composites is anticipated to play an impactful role as a significant additive for soil conditioners. Figure 4 introduces an illustrative depiction of current agricultural applications of PVA/PVP hydrogels and coatings, both in pesticides and fertilizers, and their prospective potential as soil conditioners.

## 4. Characterization

A vast amount of knowledge is available in the literature on the characterization of PVA/PVP-based hydrogels. However, the majority of publications focus on their properties in the context of medical uses [127,128,129]. This chapter provides a clear exposition of characterization tools aligned with agricultural uses. FTIR–ATR spectroscopy constitutes a fundamental technique for identifying functional groups, such as those indicative of the typical cross-linking in hydrogels, as well as the presence of entrapped pesticides or fertilizers. In addition, this technique has the capability to unveil unreacted precursors or undesired side reactions [10,65]. When assessing the soil conditioning capability, several pivotal equations are employed to ascertain a hydrogel’s polymer composition and water content, which influence water swelling and retention properties. The gel content signifies the proportion of polymers within the loaded hydrogel relative to its total mass. On the other hand, the swelling degree or water absorbance refers to the ratio of polymer water uptake (at equilibrium) with respect to its initial dehydrated state [130,131,132].

These properties can be monitored over time to obtain the swelling kinetics of hydrogels, which can be ascribed to the process of water molecule diffusion into the polymer network, which continues until it attains an equilibrium state, while the time required for this process to occur is assessed. The diffusion mechanism of small molecules into hydrogels can be elucidated using the following relationship, as developed by Korsmeyer: $Kt^{n}=\frac{M_{t}}{M_{\infty }}$. In this context, ‘M_t_’ signifies the weight of water absorbed at a specific time ‘t’, ‘M_∞_’ corresponds to the weight of water absorbed at equilibrium, ‘K’ stands for the kinetic constant, and ‘n’ serves as the diffusional exponent. The diffusional exponent ‘n’ provides insight into the diffusion, transport, or release mechanism at play in the system. Three fundamental diffusion scenarios can be employed to describe the water uptake behavior in hydrogels:

1. Fickian diffusion: In this case, the rate of transport is notably slower than the relaxation of the polymer chains. In such a scenario, the primary limiting factor affecting the swelling of the polymer is the diffusion of water into the polymer network;
2. Case of rapid water diffusion: In contrast, here, the diffusion of water occurs at a significantly faster rate compared to the relaxation process of the polymer network. In this situation, the limiting factors for water movement are primarily associated with the polymer relaxation process and the constraints imposed by the polymer network’s swelling capacity;
3. Non-Fickian or anomalous diffusion: This scenario represents an intermediate case where the diffusion of water and the relaxation rates of the polymer network are approximately equal in magnitude. It is characterized by a more complex interplay between diffusion and relaxation, leading to behavior that deviates from the typical Fickian diffusion model.

These three diffusion cases provide valuable insights into the water uptake behavior of hydrogels under different conditions and help elucidate the underlying mechanisms at play. Regarding PVA/PVP hydrogels, it is observed that the water absorption mechanism is predominantly governed by pseudo-Fickian diffusion. This behavior is characterized by water absorption curves that resemble Fickian diffusion curves; however, the attainment of the final equilibrium state occurs at a notably slower pace. In other words, while the initial stages of water absorption may resemble Fickian diffusion, the hydrogels’ water uptake eventually reaches equilibrium more gradually than what would be expected in a strictly Fickian diffusion process [133,134,135].

Water retention quantifies the alteration in water weight when hydrogels are pre-mixed within the soil. The objective of engineered soil conditioning hydrogels is to achieve a low gel content, along with a high swelling degree and water retention capacity. This combination results in the accumulation of a substantial water volume, which gradually diminishes over time. This phenomenon holds the potential to provide an exceptional irrigation resource for plants [119].

The definition, adjustment, and characterization of the mechanical properties of these hydrogels are pivotal aspects of their functional enhancement. Whether they are employed as beads within soil or designed to support seedlings, both scenarios necessitate the ability to endure various loads and pressures while retaining their structural integrity and shape [136,137]. Consequently, tests such as tensile strength and elongation at break tests are conducted to ensure compatibility with the required levels of strength [81,138,139]. As these hydrogels were intentionally crafted into different shapes intended for agricultural products, their mechanical properties such as tensile strength and particularly rheology, including viscosity and viscoelasticity, assume a significant role. In various fields, investigations involving the composition of PVA/PVP hydrogels were conducted, incorporating mechanical tensile strength and rheology measurements carried out with a universal testing machine and rheometer, respectively. Shear rate tests were employed to characterize shear behavior. Dynamic rheological studies were performed to ascertain the linear viscoelastic range (LVR) of the hydrogels, encompassing amplitude sweep tests and strain sweep measurements. The primary factors influencing the rheological properties of the hydrogels were the composition of PVA/PVP and the number of freeze–thaw cycles. The viscosity results suggest that polymer solutions containing PVA/PVP exhibit higher viscosity compared to pure PVA, primarily due to the formation of strong hydrogen bonds, electrostatic interactions, and entanglement between the compatible PVA and PVP compositions. When prepared in high-gel-content solutions, these polymers exhibit a Newtonian shear flow behavior, while those in low-gel-content solutions display a shear-thinning nature. The non-Newtonian behavior of diluted solutions is a well-established phenomenon, attributed to hydrodynamic interactions and chain extensibility [140]. These solutions can maintain their properties even under high shear rates. The strain and frequency sweep measurements confirmed the stable viscoelastic behavior of this polymer combination, attributed to the presence of both G’ and G’’. This combination promotes excellent flow properties, resulting in the formation of elastic hydrogels. An increase in the number of freeze–thaw cycles led to higher values for both G’ and G’’, attributed to the formation of stronger networks. As the PVP ratio increases (up to 6 W%), the hydrogels transition towards a more solid-like gel form, gaining enhanced mechanical tensile strength and water retention capabilities, albeit with a lower elastic modulus. However, when PVP increased above this ratio, the mechanical strength declined. This transformation arises from the creation of new cross-linked sections between PVA and PVP. In general, these hydrogels are highly flexible and robust and capable of being stretched, curled, folded, and poked. They can quickly return to their original state after the removal of an external force, indicating excellent resilience. Additionally, the hydrogels can accommodate local stress concentration and have excellent puncture resistance, making them able to withstand inhomogeneous deformation. Specifically, a rectangular-shaped hydrogel can bear a load of 100 g without breaking or cracking, demonstrating its toughness [141,142,143,144]. These properties of flowability, elasticity, and mechanical strength are essential for molding hydrogels into desired stable agricultural shapes.

The physical properties of PVA/PVP hydrogels, such as the degree of crystallinity and miscibility, were determined through DSC analysis. The degree of crystallinity was found to be significantly influenced by the ratios of PVA to PVP; higher PVA concentrations led to intensified PVA crystal regions. DSC analysis also demonstrates a high level of miscibility between PVA and PVP, indicating their cohesive and integrated properties [130]. This good miscibility was also substantiated by morphology analyses using E-SEM and AFM, which reveal a uniform surface texture and roughness, respectively. AFM and E-SEM are also valuable for characterizing the shapes, sizes, and distribution of entrapped pesticides or fertilizers, along with their morphological impacts on the surface of the hydrogels [10,65].

To further quantify the entrapped pesticides or fertilizers, a range of analytical methods can be employed. The choice of method depends on the specific characteristics of the analyzed agent and the method’s specifications. As an example, if the entrapped pesticide undergoes a well-defined redox process like HP, its quantification can be achieved through titration with potassium permanganate. However, in the case of an entrapped pesticide with strong UV absorption, such as thymol, quantification can be accomplished by measuring absorption using a UV spectrophotometer.

In scenarios involving entrapment of multiple agents within the hydrogel matrix, it is imperative to address factors such as reagent selectivity and the precise sequencing of procedural steps. For instance, consider a scenario where both thymol and HP are entrapped within a hydrogel. In this case, using a general oxidizer such as potassium permanganate would lead to oxidation of both agents without differentiation. To address this issue, specific indicators such as peroxide sticks may be employed initially to detect the presence of HP. Thymol quantification can be conducted in a sequential manner [65].

The assessment methodology for fertilizers must align with the unique characteristics of the given scenario. When aiming to identify the nitrogen source originating from urea, which is encapsulated within PVA/PVP copolymeric coatings, the proven Kjeldahl method stands out as the most appropriate choice. After determining the content of the entrapped substance, a systematic process of sequential sampling over specific intervals enables the construction of a release profile graph. This graph serves as a fundamental tool for comprehending the way a pesticide or fertilizer is gradually released into a plant’s surrounding environment. Hence, the release profile of the hydrogels can be fine-tuned to match the specific needs of plants [10,80,118].

Thermal stability is an additional crucial factor. Many pesticides or fertilizers demonstrate limited stability in their pure form. For example, thymol grains have a substantial vapor pressure, making them volatile and leading to swift evaporation over a short period [114,145]. HP naturally decomposes into oxygen and water over time or is rapidly flushed away when dissolved in water [113]. Urea is another example; due to its instability in the presence of water, it undergoes rapid hydrolysis and subsequent flushing [146,147]. Consequently, evaluating the role of these hydrogel casings in maintaining the stability of entrapped agents can elucidate their significance in preserving these agents for extended durations, especially under demanding conditions such as dynamic elevated temperatures in TGA–MS, within a pre-heated isotherm incubator, or during exposure to rapid water flushes [65,118].

The forthcoming discussion involves a comprehensive examination of the influence of hydrogels on biological activity concerning their specific targets. Hydrogels, which are formulated to enhance plant fertilization and irrigation, are assessed using inductive approaches. This evaluation encompasses an analysis of various plant growth metrics, including measurements of fresh and dry biomass, stem, and root elongation, as well as quantification of the proportion of total nitrogen content within leaves and the dehydrated plant components [118,148]. A primary objective involves eradicating or preventing proliferation of plant pests while also assessing the effectiveness of biocidal agents.

This evaluation can be conducted by direct measurements of the pests themselves or indirectly by quantifying the observable signs of their activity on the host plants. The studies we reviewed focusing on PVA/PVP hydrogels infused with pesticides showcased the application of these assessment techniques against various pathogens [10,65].

An important consideration pertains to the complete life cycle of these hydrogels, including preventing their accumulation and the potential environmental risks that might emerge over time. This achievement is made possible through the biodegradation of PVA/PVP compounds [130]. Several complementary methods are employed to evaluate the biodegradability of these compounds. Soil burial is a commonly used technique for degradation assessment. It involves placing hydrogels in soil under controlled conditions that simulate natural degradation processes. The hydrogels’ dry weight loss over time is measured, and the difference in weight is considered as the portion that undergoes biodegradation [117]. To validate the biodegradation, the qualitative clear-zone method is employed as a straightforward visual technique. This method involves observing a distinct, clear area around microbial colonies. This clear zone is attributed to the enzymatic activity of microorganisms, leading to the dissolution of the surrounding area. Furthermore, to quantify and identify microbial degradation, the plate screening method is utilized. This technique involves isolating, identifying, and ranking bacteria based on their contribution to the largest measured solubilized margins [130].

## 5. Conceptual Shapes of PVA/PVP-Based Hydrogels in Crops

Various techniques are viable for generating a range of applications using hydrogels. One of the most widely used and scalable techniques for practical applications is solution casting. This process involves pouring the bulk solution into an inert mold of the desired shape or using a doctor blade to cast a thin film. This method is straightforward, allows for easy adjustment of film thickness, is cost-effective, and can be employed with various polymer components to produce homogeneous castings. Nonetheless, if a high level of mechanical strength is required, this method may not be adequate [149,150,151]. Injection molding is a molding technology that involves melting polymers using a screw and an external heating device, followed by injecting the molten material into a mold to produce the desired product as the mold cools. This process is repetitive, allowing for rapid production, and it is compatible with both pure and composite materials. Injection molding can create complex geometries with high precision. However, this method does face certain challenges, including limitations in terms of suitable materials with appropriate rheological properties, process complexities, and equipment-related challenges [152,153,154]. Compression molding is a closed molding process wherein raw materials are placed within a cavity mold under controlled heat and pressure conditions to create a range of composite products known for their relatively high mechanical strength. The preparation of raw materials and the compression molding process, including specified applied pressures, heat levels, and durations, are critical parameters that significantly influence the mechanical performance of the final product. This method is highly reproducible and widely adopted in industrial settings due to its cleanliness, cost-effectiveness, efficiency, and the fact that it can be performed without solvents. However, because it necessitates pretreatment of raw materials and involves multiple variables such as compression force, heat, and duration, achieving optimal working conditions may require thorough investigation and optimization [155,156,157]. Three-dimensional printing represents the most modern technique among the four mentioned. It can be categorized into four primary printing methods:

1. Extrusion printing: In this method, continuous filaments serve as the building blocks;
2. Inkjet printing: This technique utilizes low-viscosity inks, often combined with in situ or post-fabrication processing to achieve mechanically stable structures;
3. Stereolithography printing: Stereolithography employs photopolymerizable prepolymer solutions;
4. Laser-assisted printing: Laser beams are employed to construct intricate structures from ink droplets.

The primary advantage of 3D printing is its ability to create complex hierarchical multi-material structures at a reduced cost. Additionally, these printers are user-friendly and can be quickly adjusted to meet specific parameters. However, it is essential to prepare printing formulas with precise rheological specifications to ensure the desired mechanical structure. Furthermore, it is worth noting that, as of now, these printers have not yet reached the levels of industrial output achievable by the traditional shaping methods previously mentioned [158,159,160].

**Figure 5:**
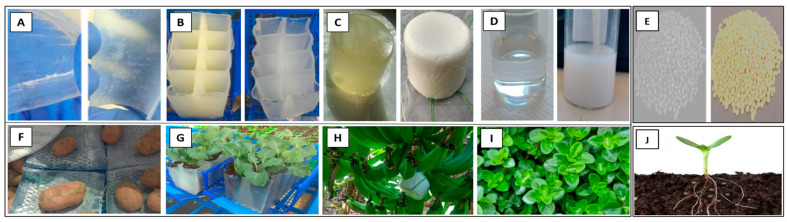
PVA/PVP-based hydrogels composed of 15 w% PVA and 6 w% PVP engineered into various forms for plant and crop protection: neat and loaded with 6 w% HP coatings on polymeric sheets (**A** **left** and **right**, respectively), implemented as potato crop bags stored in 2–10 °C refrigerated room (**F**). Seedling plates unloaded and loaded with HP (**B** **left** and **right**, respectively), for implanting seedlings placed in greenhouse (temperature: 18–24 °C, relative humidity: 40–80%). (**G**). Capsules loaded and unloaded (**C** **left** and **right**, respectively) with 5% thymol (*w*/*w*) anchored on banana plantations are protected by net sheets and grow in mediterranean weather all year (**H**). Spray formulation, unloaded and loaded with 5 w% thymol (**D** **left** and **right**, respectively) utilized on seedling foliage placed in greenhouse (**I**); uncoated and coated encapsulations of fertilizer granules (**E** **left** and **right**, respectively) implemented in plant soil placed in greenhouse (**J**).

One approach involves creating uniform shapes that are laden with fertilizers and pesticides. This section introduces specific configurations, including hydrogel coatings infused with HP (Figure 5A,F). These coatings are produced by applying hydrogel precursor solutions onto polymeric sheets using the Mayer rod technique [65,161]. The outcome is the production of coated sheets, facilitating the creation of an atmosphere saturated with active ingredient vapor or direct contact with the crops (Figure 5F). In cases where a bulk form is required, castings are produced by pouring the hydrogel precursor solutions into a designated mold with the desired shape [10]. The resulting castings manifest as hydrogel seedling plates infused with HP and urea (Figure 5B,G) or as capsules loaded with thymol (Figure 5C,H). These structures possess the capability to release active substances into the root systems of plants or emit fumes that envelop fruits, depending on the specific configuration.

An additional form comprises liquid hydrogel formulations derived from diluted PVA and PVP precursor solutions incorporated with HP and thymol. These formulations are specifically designed for direct spraying applications. The resultant solutions are applied onto the foliage of seedlings, forming a protective coating (Figure 5D,I). An additional form of encapsulation of urea granules constitutes an alternative technique for developing safeguarded fertilizers, mitigating issues related to swift decomposition, leaching, or evaporation (Figure 5E,J). This encapsulation methodology can be achieved by utilizing a sugar-coating machine or through the application of a hot coat sprayer apparatus [162].

## 6. Recent Agricultural Applications and Results with PVA/PVP-based Hydrogels

A plethora of studies extensively investigated the individual applications of PVA and PVP within an agricultural context [52,163,164]. Nevertheless, the amalgamation of PVA and PVP (PVA/PVP) for agricultural purposes remains relatively novel. As a result, there is a lack of comprehensive works in the literature on these hydrogels and their derivatives. The present review encompasses a range of studies, including both our recent contributions and research conducted by other groups. A recent study introduces the development of Nervilia fordii extract-loaded electrospun PVA/PVP nanocomposites for antioxidant packaging [130]. This innovative approach holds the potential to revolutionize agrifood preservation by providing nanofibers with a significantly increased surface area, leading to superior spatial antioxidant release compared to traditional bulk hydrogels. Furthermore, the resulting morphology of these fibers remained unchanged when loaded with antioxidants, in contrast to the discussed loaded hydrogels, which exhibited significant morphological changes compared to their unloaded counterparts. Both our work and the aforementioned study offer efficient loading methods, improved thermo-stability of active substances, and promising results for applications in preserving tested products.

These studies shed light on the various pesticidal capabilities of these hydrogels and their derivatives against crop viruses and fungi [4,32]. Additionally, these hydrogels function as carriers for fertilizers with promising results for plants growth [39,41,63], enabling their controlled and gradual release. A correlated and advantageous outcome of hydrogel utilization, which holds true in this specific case as well, is the capacity to retain water and subsequently release it over a period. This stands in contrast to scenarios involving irrigation without the incorporation of hydrogels into soils, where water tends to seep rapidly, leading to its loss. Hence, it is conceivable that this could serve as a potential enhancer for soil conditioning. The applications were executed through diverse preparations and formulations of the PVA/PVP composition, tailored to suit various purposes (Table 1).

**Figure 6:**
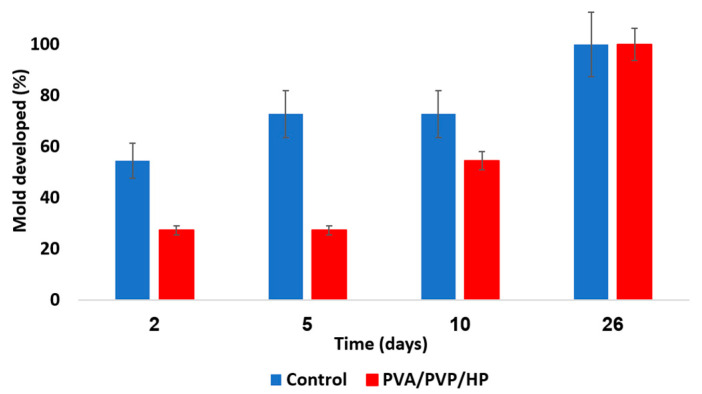
Mold growth rate over several days. Uncoated PE sheets (blue) are a control. PVA/PVP/HP hydrogel coated sheets (red) contain an entrapped anti-mold component.

**Figure 7:**
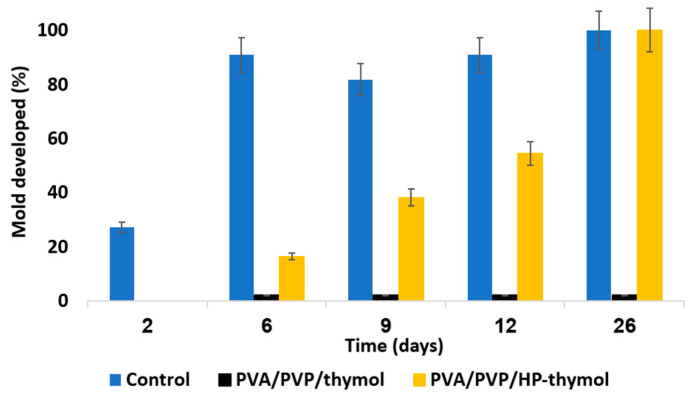
Mold growth rate over several days as a function of coating type. The PVA/PVP hydrogel PE coated sheets (blue) are a control. PE sheets coated with PVA/PVP/thymol (1.25 w% thymol precursor, black) or PVA/PVP/HP-thymol (0.63% each of HP and thymol precursors, orange) present coatings with entrapped anti-mold components.

**Figure 8:**
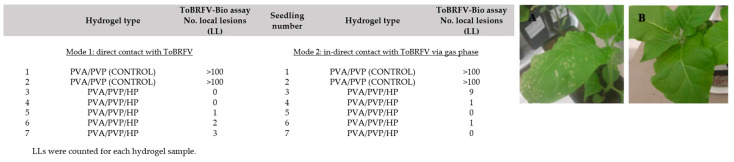
Biological assay of ToBRFV on Nicotiana tabacum. 35 (**A**) Control tobacco plants (control treatment with no active molecule) developed symptoms of local lesions (LLs) on leaves following ToBRFV infection. (**B**) No ToBRFV LL symptoms were developed following hydrogen peroxide-loaded hydrogel treatment.

**Figure 9:**
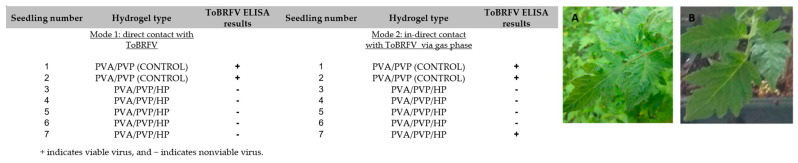
Biological assay of ToBRFV on tomato plants. (**A**) Seedlings infected with ToBRFV after being inoculated in a control PVA/PVP hydrogel. (**B**) Tobacco seedling infected with the virus after being inoculated in a PVA/PVP/HP hydrogel.

Representative results from our recent studies demonstrated the effective results of these hydrogels against various pests as shown in Figure 6, Figure 7, Figure 8 and Figure 9, respectively [65,116].

## 7. Sustainability

Conventional agricultural methods involve extensive utilization of water, fertilizers, and pesticides. However, a significant portion of these resources go to waste due to processes such as seepage, evaporation, and rapid decomposition. This accumulation of residues over time has a notable adverse impact on the surrounding environment [185]. Hydrogels, especially those composed of PVA and PVP, exhibit exceptional capacity in terms of stability, retention, and controlled release of water, fertilizers, and pesticides [10,65,118]. Consequently, broadening their application presents a viable alternative for resource preservation. PVA/PVP hydrogels, particularly in their physically cross-linked state, demonstrate convenient reversibility. Application of mild heat is adequate to dissolve them back into their initial precursor solutions [186]. These solutions possess the capacity to undergo both regelation and reloading procedures. These inherent properties significantly enhance their potential for repeated utilization, resulting in a decreased demand for raw materials or active ingredients [187]. Regarding chemically cross-linked PVA/PVP hydrogels, the covalent bonds formed within these hydrogels are of a permanent nature and exhibit irreversibility [188]. Nevertheless, these rigid structures acquire enhanced capability for retaining and prolonging the release of active substances, such as pesticides Figure 3.

Several parameters exert influence over biodegradation. Previous studies conducted long ago provide insights into the separate biodegradation characteristics of PVA and PVP components, enhancing our understanding of their respective degradation behaviors [88,189]. PVA has a well-known biodegradation mechanism and, which exhibits favorable results [190], while PVP shows relatively high resistance due to its pyridine cycle, which is considered relatively inert and less susceptible to microbial digestion. Subsequent investigations addressed the biodegradation of combined components. The influence of the PVA-to-PVP ratio on this process was examined, revealing that as PVP becomes more predominant, the extent of biodegradation decreases [130]. This outcome is consistent with the findings of earlier studies focused on investigating each component individually. Both the type and intensity of cross-linking are parameters that directly influence the rate of biodegradation. Chemical covalent cross-linking is notably more stable compared to physical cross-linking, which confers greater resistance to microbial degradation. Furthermore, whether achieved through physical or chemical cross-linking, increasing the density of cross-linking leads to heightened resistance against biodegradation in both cases [191].

Microbiome composition is an additional parameter, and the isolation of various bacteria involved in the degradation process reveals that the dominant microbe responsible for PVA/PVP degradation is the *Pseudomonas putida* strain. The microbe’s prosperity depends on the soil type in which the hydrogels are buried. Various soil types possess unique compositions and physical properties, leading to diverse ground conditions that can influence the inhibition of different microbiomes. Accordingly, a study effectively illustrated the impact of rich clay soil containing a dense microbiome, which expedited the biodegradation of PVA/PVP hydrogels. In contrast, when comparable hydrogels were introduced into nutrient-poor sandy soils, they exhibited inferior levels of biodegradation activity [24]. This description pertains to the biodegradation of the hydrogel casing.

Another crucial aspect involves the material released by biodegradation. Both pesticides and fertilizers are distributed to cover the surroundings of plants, with only limited amounts taken up by the plants themselves. The remaining residues have the potential to pose environmental risks [185,192,193]. Consequently, numerous efforts are focused on identifying environmentally friendly alternatives. An example of such an alternative pesticide is HP, which undergoes spontaneous decomposition over time, yielding oxygen and water. As a result, no toxic residues are left behind [194]. Another viable alternative is the use of the natural essential oil thymol. Thymol either exhibits negligible residue formation, as it undergoes rapid photodegradation when exposed to sunlight and water, or degrades when buried in soil. A significant portion of thymol tends to dissipate prior to degradation, attributed to its high volatility [70,195]. Our recent investigations showcase PVA/PVP hydrogels entrapping diverse ratios of HP and thymol, which exhibit remarkable biocidal efficacy against Tomato Brown Rugose Fruit Virus (ToBRFV) and mold [10,65].

**Figure 10:**
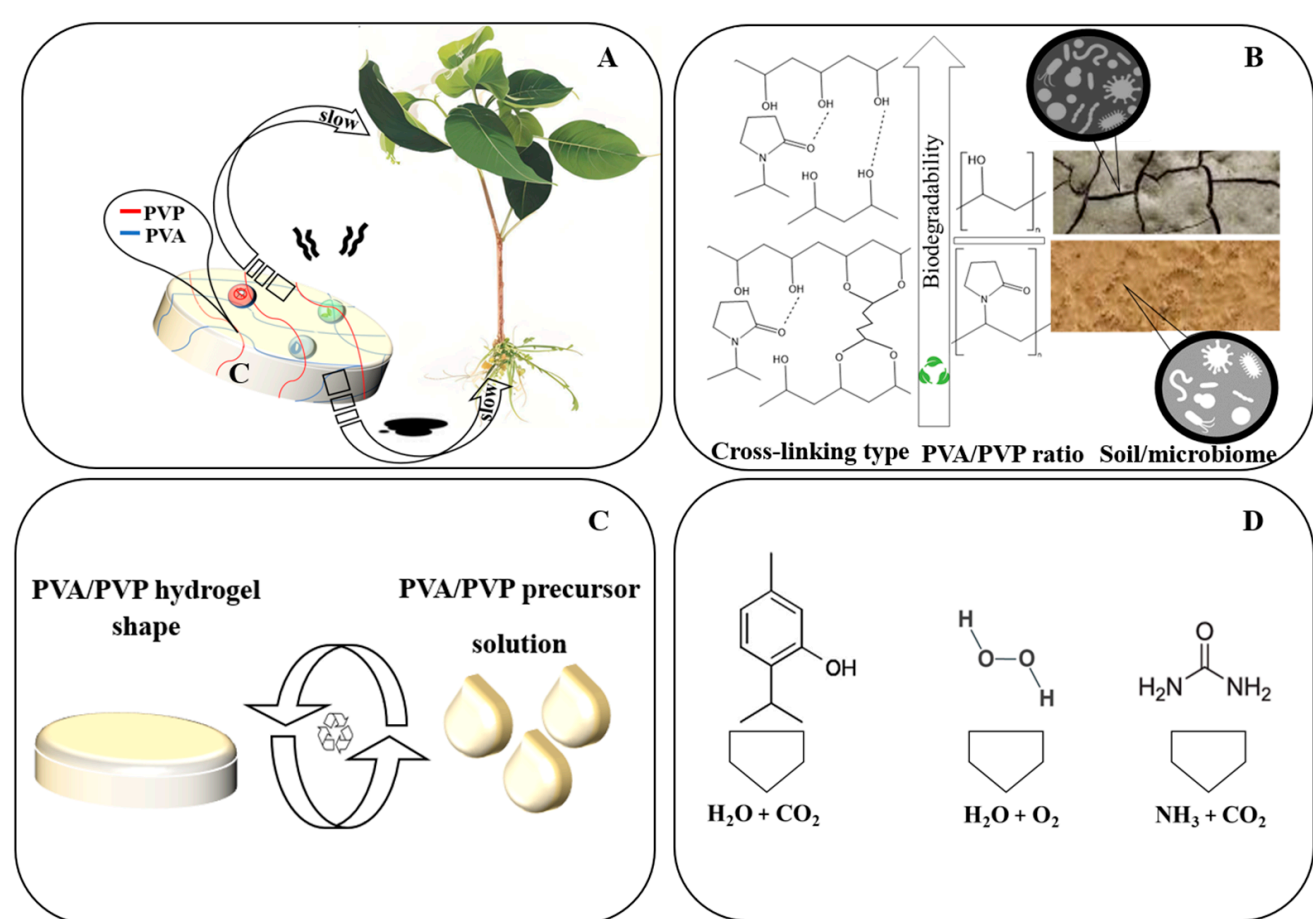
(**A**) Slow release utilizing PVA/PVP hydrogel, making pesticide, fertilizer, and water application to plant foliage and roots more efficient while minimizing waste. (**B**) Parameters can influence the biodegradation of hydrogels, with the goal of achieving greater biodegradability. (**C**) Reversible physical cross-linked hydrogels allow for reusability with less waste. (**D**) Degradation of released pesticides and fertilizers results in relatively environmentally friendly residues.

We conclude by addressing the sustainability of fertilizers in relation to PVA/PVP hydrogels, with a specific focus on nitrogen, a pivotal element for plant growth. Urea, a prevalent nitrogen source, is widely recognized as a biodegradable plant nutrient. Plants can directly assimilate urea through their foliage or indirectly acquire ammonium and nitrate ions, byproducts of urea biodegradation, through their roots. This process takes place through the enzymatic activity of urease present in the soil microbiome, facilitated by the presence of water. Urea undergoes rapid hydrolysis, producing ammonia, which is readily available for plant uptake [146,196]. A comprehensive study was conducted to examine the effects of encapsulating urea with PVA/PVP coatings on the growth of Chinese kale plants. These coatings effectively minimized ammonia evaporation, providing a more sustained and favorable supply of ammonia for plant uptake. Consequently, plants treated with encapsulated urea exhibited enhanced development and a healthier appearance compared to those exposed to conventional urea granules [118]. Figure 10 visually represents these sustainability aspects.

## 8. Conclusions and Outlook

Agricultural practices have undergone progressive industrialization and intensified utilization of fertilizers, pesticides, and irrigation systems to fulfil the escalating global food demand. There is a necessity to establish regulatory frameworks and optimization strategies governing the precise allocation of these resources in alignment with the requisite nourishment of plants, considering both environmental sustainability and economic prudence. Hydrogels are recognized for their potential to effectively fulfil plant needs while also preserving the environment and valuable resources. PVA/PVP hydrogels exhibit well-known characteristics such as extended release of essential substances and possible controlled irrigation of plants. What sets this combination apart is its unique ability to create various shapes using only physical cross-linking in many applications, which is more environmentally friendly compared to chemically cross-linked hydrogels. Nonetheless, the hydrogels can potentially be enhanced through chemical cross-linking if an extra level of strength is desired. Adjustment of the PVP ratio allows for regulation of water absorption and transmissivity, enhancing compatibility with a wide range of entrapped chemical compounds.

Hence, PVA/PVP combination provides a convenient way to adjust these hydrogels for diverse agricultural applications. These hydrogels were suggested to be synthesized using various preparation techniques, which encompass methods such as irradiation, chemical cross-linking, and physical cross-linking via freeze–thaw cycles. The choice of method depends on the desired properties of the hydrogels. Subsequently, these properties were evaluated from both physical and chemical standpoints, and their impact on biological activity was investigated.

The reviewed studies effectively demonstrate the advantageous attributes of PVA/PVP-based compositions across various applications in the realm of plants and crops. This lends support to the potential of extensive utilization of such compositions in agricultural products. Nonetheless, several challenges persist. PVP’s resistance to degradation results in its persistence and accumulation. Conventional polyolefins exhibit considerably lower costs in comparison to PVA and PVP. Moreover, chemical or irradiation-cross-linked reinforcement of PVA/PVP-based hydrogels diminishes their susceptibility to biodegradation. These cross-linking methods are also occasionally prone to side reactions and can cause the deterioration of entrapped substances.

Considering the challenges, practical and commercial implementation of PVA/PVP- based agricultural applications could be facilitated by achieving a delicate equilibrium between the extent and nature of cross-linking with a preference for physical cross-linking. In addition, exploring the feasibility of reusing hydrogels for successive activity cycles by replenishing with fresh portions of active substances may prove advantageous. Moreover, employing minute quantities of additives such as thin coatings or enhancers for soil conditioners could be a viable approach for mitigating consumption of raw materials.

## Figures and Tables

**Table 1:** Hydrogels and their PVA/PVP derivatives, designed for agricultural applications or possessing properties suitable for agricultural use.

Hydrogel Type	Application Method	Purpose	Reference
Physical cross-linked PVA/PVP loaded with hydrogen peroxide	Loaded hydrogels in direct contact with the virus or exposure via released vapor	Virucide: eradication of Tomato Brown Rugose Fruit Virus (ToBRV)	[10]
Physical cross-linked PVA/PVP loaded with varied thymol and hydrogen peroxide ratios	Hydrogel coatings placed on top of hay	Fungicide: prevention of mold growth on hay	[65]
Physical cross-linked PVA/PVP	Coatings encapsulated urea granules	Slow-release fertilizer and soil conditioner	[118]
Chemical cross-linked PVA/PVP with epoxy resin and zeolite	Membrane slow release of urea	Slow-release fertilizer	[81]
Chemical cross-linked with biochar copolymer PVA/PVP	Coating material encapsulates urea granules	Slow-release fertilizer	[117]
Physical cross-linked Chitosan/PVA/PVP	Films loaded with chitosan nanoparticles	Fungicide: prevention of fungus growth on strawberry	[165]
Chemical cross-linked PVA/PVP	Films loaded with plant extracts	Broad-band antimicrobial activity	[166]
Physical cross-linked PVA/PVP	Coatings encapsulate urea	Slow-release fertilizer	[167]
Physical cross-linked PVA/PVP	Films loaded with cyanine derivatives and their C_u_^2+^ complexes	Broad-band antimicrobial activity	[168]
Physical cross-linked PVA/PVP/Glycerol	Coated seeds loaded with pro-microbial inoculant	Plant growth-promoting bacteria	[169]
PVA/PVP/Cellulose	Films loaded with ZnO and cellulose	Antimicrobial activity	[170]
Physical cross-linked PVA/PVP	Films loaded with ZnO:Fe modified with vitamin C nanoparticles	UV protection and antimicrobial activity	[171]
Physical cross-linked PVA/PVP	Films loaded with anthocyanin	UV and visible radiation protectant	[172]
Chemical cross-linked CMC/PVA/PVP	Nanofiber mats	Moisture reducer: fruit and vegetable preservatives	[173]
Physical cross-linked PVA/PVP/PEG	Coatings loaded with celery leaf extract on aluminum foil	Insect repellent	[174]
Chemical cross linked PVA/PVP	Microneedles loaded with epsilon-poly-L-lysine	Reduction of fungal infections in citrus fruit pericarp	[175]
Physical cross-linked PVA/PVP	Protective solution for the preparation of silver nanoparticles	Antimicrobial activity	[176]
Physical cross-linked PVA and PVP	Coated seeds loaded with pro-microbial inoculant	Plant growth-promoting bacteria	[177]
Physical cross-linked PVA/PVP	Films loaded with CuO	Antimicrobial activity	[178]
Physical cross-linked PVA/PVP	Films loaded with OrmocarpumCochinchinense Leaf Extract	Antifungal and antimicrobial activity	[179]
Physical cross-linked PVA and PVP	Encapsulation of living bacteria in dry coatings	Bioremediation	[180]
Physical cross-linked PVA and PVP	Nanofibers loaded with hormones	Plant growth-promoting hormones	[181]
Physical cross-linked PVA/PVP	Nanoribbons loaded with magnesium oxide	Soil enrichment	[182]
Physical cross-linked PVA/PVP	Nanofibers	General use in agriculture	[183]
Physical cross-linked PVA/PVP	Nanocomposite films loaded with crystalline nanocellulose	Food packaging	[184]

## Data Availability

The data presented in this study are openly available in article.
